# Advancing Medulloblastoma Therapy in Pediatrics: Integrative Molecular Classification and Emerging Treatments

**DOI:** 10.3390/brainsci15080896

**Published:** 2025-08-21

**Authors:** David T. Kim, Michaela Uloho-Okundaye, Stephen C. Frederico, Santosh Guru, Min J. Kim, Steven D. Chang

**Affiliations:** 1 Harvard College, Harvard University, Cambridge, MA 02138, USA; davidkim@college.harvard.edu (D.T.K.); michaelaokundaye@college.harvard.edu (M.U.-O.); 2 Department of Neurological Surgery, University of Pittsburgh School of Medicine, Pittsburgh, PA 15213, USA; stephen_frederico@hms.harvard.edu; 3 School of Clinical Medicine, University of Cambridge, Cambridge CB2 0SP, UK; sg928@cam.ac.uk; 4 Department of Neurosurgery, Harvard Medical School and Mass General Brigham, Boston, MA 02115, USA; mkim85@bwh.harvard.edu; 5 Department of Neurosurgery, Stanford School of Medicine, Palo Alto, CA 94304, USA

**Keywords:** medulloblastoma, brain tumors, pediatric brain tumors, targeted therapy, oncology, pediatric oncology, immunotherapy, precision medicine, WHO CNS5

## Abstract

Medulloblastoma (MB), the most common malignant pediatric brain tumor, has undergone reclassification from a histologically defined disease to a genetically stratified spectrum of distinct subgroups: WNT, SHH, Group 3, and Group 4. Advances in molecular profiling, as captured in the 2021 WHO CNS5 classification, have shown meaningful heterogeneity in terms of tumor biology, prognosis, and therapeutic response. However, translating these insights into precise, less toxic treatments remains an ongoing challenge. This review synthesizes current knowledge on MB subgroup biology, treatment strategies, and emerging therapies such as subgroup-specific inhibitors, immunotherapies, and novel chemotherapeutic regimens. This review also explores risk-adapted approaches while addressing global disparities in access to diagnostics and care. As the field moves toward individualized medicine, closing the gap between molecular understanding and equitable implementation will be crucial to improving outcomes and quality of life for children with medulloblastoma worldwide.

## 1. Introduction

Medulloblastoma (MB), an embryonal tumor of the cerebellum, is the most common malignant pediatric brain tumor, accounting for nearly 20% of all pediatric CNS neoplasms. Among pediatric tumors, embryonal tumors occur throughout childhood development but are most prevalent early in life; children aged 1 to 9 years old display a fivefold to tenfold higher incidence than adults. Childhood MB represents approximately 40% of all posterior fossa tumors and is among the most prevalent pediatric embryonal tumors [1]. Table 1 summarizes the numbers of cases reported in the United States between the years of 2017 and 2021.

First distinguished from glioma by Bailey and Cushing in 1925, MB was formerly termed as a primitive neural ectodermal tumor (PNET) and consists of small blue cells [2]. Its progression is often rapid, and MB cells tend to disseminate through the cerebrospinal fluid (CSF) to distant foci in the brain and spinal cord. The presentation can be non-specific, with features such as fatigue, nausea, vomiting, and behavioral change, or consist of alarming features, prompting emergent neuroimaging, such as increasing head circumference with tense fontanelle, intractable vomiting, eye deviation, developmental regression, focal neurological deficits, and/or reduced mental status. The initial workup consists of a full neurological examination, full neuroimaging including MRI of the brain and spine with and without contrast, and consultation with neuro-oncology and neurosurgery. Initial management is dependent on the presence of hydrocephalus and/or mass effect of the lesion. Diversion strategies (e.g., external ventricular drain, endoscopic third ventriculostomy) may be required emergently to facilitate maximal safe admission, the completion of workup, and maximal safe operative resection. After operative resection of the lesion, histopathological diagnosis is confirmed and in centers capable of molecularly analyzing tumors, tissue is sent for molecular profiling. The 2007 WHO Classification of Tumors of the Central Nervous System update categorized MBs into 5 main subtypes, consisting of classic, large-cell, anaplastic, desmoplastic nodular, and MBs with extensive nodularity (MBEN) [2]. Since then, there have been various updates to MB classification integrating the breadth of molecular data from the basic science community. The most recent WHO Classification of Tumors of the Central Nervous System was made available in 2021 [3], integrating the suggestions for updates made by the Consortium to Inform Molecular and Practical Approaches to CNS Tumors (not officially WHO) (cIMPACT-NOW).

### Classification of MB Subgroups

With the advances in targeted immunotherapies for childhood leukemias improving outcomes, in 2014, pediatric brain tumors became the leading cause of childhood cancer-related mortality [4]. Over the past decade, the immense efforts of the clinical and scientific community have contributed to pediatric MB becoming the most sequenced and studied pediatric solid tumor entity [5]. Consolidating the discoveries from the basic science and clinical research efforts, updates to the 2021 edition of the WHO Classification of CNS tumors were completed by the cIMPACT-NOW group. While our understanding of the molecular basis of pediatric MB has increased dramatically, we have yet to translate this knowledge into significant advances in the management of childhood MB. In the 2021 WHO Classification of CNS Tumors 5th edition (WHO CNS5), the histopathological classification remains, and to this, the authors have added the molecular classification detailed in their WHO CNS5 publication [3]. While this update did not have a corresponding cIMPACT-NOW document featuring the molecular classification of MB, as occurred for ependymomas or gliomas, the evidence available from prominent research in the field was summarized and integrated into WHO CNS5. In the 5th edition, guidance on distinguishing tumor subtypes was provided through molecular markers instead of histology alone, refining the diagnostic process, and stratifying pediatric brain tumors by genomic and epigenomic features in addition to microscopic morphologies and histological characteristics. Over time, with advances in genomic and molecular profiling, classifications have evolved beyond traditional histology alone to incorporate molecular subgrouping, significantly refining diagnosis and prognosis.

The WHO CNS5 integrates 4 molecular groups. These are WNT-activated, Sonic Hedgehog (SHH)-activated (divided on the basis of TP53 status), Group 3, and Group 4, comprising non-WNT/non-SHH MBs [3]. According to new knowledge of biological heterogeneity discovered through further transcriptome profiling and large-scale methylation, it is now understood that there are four subtypes of SHH and eight subgroups of non-WNT/non-SHH MBs. This delineation of subtypes provides further understanding of the heterogeneous outcomes observed within childhood MB subgroups and aims to facilitate the clinical utility of molecular discoveries of pediatric MB in the era of personalized medicine [3]. WHO CNS5 has updated the initial 2016 WHO histopathological classification of MBs, including the four morphologic types of classic, desmoplastic/nodular, MBs with extensive nodularity (MBEN), and large-cell/anaplastic MBs [3]. In the WHO CNS5 update, these are now encompassed in one section describing morphologic patterns of MBs. Morphological differences in MBs are linked to specific clinical outcomes and patterns have been observed in association with molecularly defined MB. The vast majority of WNT-activated MBs are linked to classic morphology, while the majority of large-cell/anaplastic tumors are of the SHH-3 subgroups or the G3/4 subgroup 2 [3]. According to the 2021 WHO CNS5 Classification updates, MBs should be reported using a layered and integrative format inclusive of these updates. In WHO CNS5, MBs maintain a CNS WHO grade 4 designation, even with the observation that treatment can lead to a favorable outcome, as tumors are graded by their expected natural history without treatment. This change is most notable with WNT-activated MBs, which have aggressive behavior when left untreated but are responsive to therapeutic regimens and can lead to long-term survival [3]. Table 2 summarizes the differences between the WHO CNS4 and CNS5 MB classifications.

In this review, we will highlight the field’s current understanding of what drives the tumorigenesis of each subtype; current treatments and outcomes for each subtype; and what is on the horizon for future trials and discovery related to MBs. This review will focus on the integration of molecular findings into diagnostics and treatments for each subgroup of childhood MBs, emphasizing certain features such as their updated classification, molecular subgroupings, and ongoing efforts toward and particular challenges in the development of effective treatments. In the subsequent sections, we will discuss the detailed molecular classification of MBs, current standard treatments, clinical outcomes by subtype, and emerging therapies that promise personalized and improved management.

## 2. Subgroups of MB Expanded

**Figure 1:**
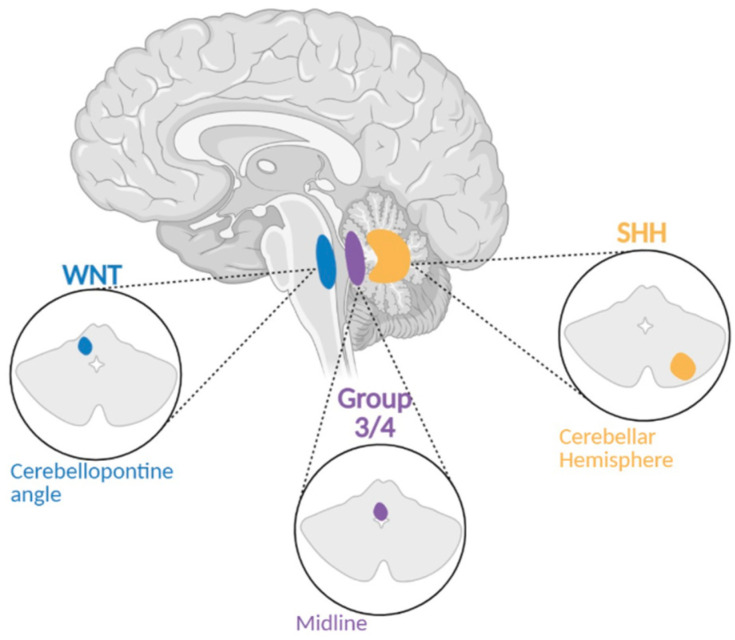
Location of Medulloblastoma by Subgroup. A figure demonstrating where subgroups of Medulloblastoma develop within the cerebellum and surroundings.

The molecular subgrouping of MBs is very important for personalized clinical management, as each subgroup presents unique genetic drivers and prognostic outcomes. In this section, we describe the molecular underpinnings and classification frameworks that define each medulloblastoma subgroup (Figure 1).

We also explicitly included the incidence of metastatic disease for each major molecular subgroup to provide critical prognostic context:

- WNT: ~10% present with metastatic disease, but prognosis remains excellent.
- SHH: Metastatic rates are variable; worse outcomes are observed, particularly with TP53 mutations.
- Group 3: ~50% of cases present with metastasis.
- Group 4: ~30% of patients present with metastatic disease.

### 2.1. Wnt Subgroup

The wingless or Wnt MB subgroup represents the most clinically favorable subgroup due to its distinctly favorable long-term prognosis compared to the other three subgroups. The majority of Wnt MBs are characterized by a mutation in the CTNNB1 gene, typically at exon 3, resulting in a lack of phosphorylation-driven degradation of β-catenin within the nucleus, leading to the constitutive activation of the Wnt signaling pathway [6]. Less commonly, the inactivation of other genes that activate the Wnt signaling pathway—such as APC, a tumor suppressor gene involved in the ubiquitination of β-catenin, causing its degradation—is identified [7]. For Wnt MBs, a cancer predisposition syndrome designated Turcot syndrome has an increased tendency to form MBs, resulting from germline mutations of the tumor suppressor gene APC, a known inhibitor of the WNT pathway [8]. Moreover, somatic mutations of CTNNB1 encoding β-catenin have been discovered in some sporadic MBs, supporting the role of the signaling pathway in the development of this tumor [9]. WNT MB generally has a favorable prognosis, as detailed further in the outcomes section.

WNT MB most commonly presents in older children, with a peak incidence between 6 and 12 years of age, and is exceedingly rare in infants. There is no clear sex predilection, distinguishing it from Group 3 and Group 4, which exhibit strong male predominance [8]. Histologically, WNT tumors are nearly always of the classic subtype [10]. One of the hallmark cytogenetic features of WNT MB is monosomy 6, present in approximately 80–90% of cases, and this is often used as a surrogate diagnostic marker [11]. The anatomical origin of WNT MB is believed to be the dorsal brainstem or lower rhombic lip, rather than the cerebellar hemispheres, which may partially account for its low metastatic propensity and excellent prognosis [11,12].

In addition to canonical CTNNB1 mutations, some WNT MBs also have mutations in DDX3X, an RNA helicase gene, although the functional implications of this co-mutation are still under investigation [13]. Recent classification efforts, particularly those by [11], have divided WNT MBs into two subtypes: WNTα and WNTβ. WNTα comprises the majority of pediatric WNT cases, is nearly universally associated with monosomy 6, and is seen primarily in children under 16. WNTβ tumors, in contrast, are more frequent in adolescents and adults and may lack chromosome 6 loss altogether. Importantly, despite this molecular divergence, there is no evidence that WNTβ confers a worse outcome than WNTα. Both subtypes show 5-year overall survival rates above 90%, placing WNT MBs within the “low-risk” category of current stratification schemes. Consequently, WNT MBs are a major focus of treatment de-escalation trials, aiming to reduce long-term neurocognitive sequelae while maintaining excellent survival.

### 2.2. SHH Subgroup

In contrast to the WNT subgroup’s favorable prognosis, the SHH subgroup demonstrates more variable outcomes and distinct molecular drivers. The SHH subgroup is named after the Sonic Hedgehog signaling pathway that drives tumorigenesis in most cases. This MB subgroup was identified by transcriptional profiling and immunohistochemical staining for Secreted Frizzled-Related Protein 1 (SFRP1) [14]. There is a predisposition to develop SHH MBs caused by Gorlin Syndrome, germline mutations in the SHH receptor PTCH. Infantile SHH MBs can be predisposed by a germline mutation in the SHH inhibitor SUFU as well. Finally, somatic mutations of PTCH, SMO, and SUFU, coupled with amplifications of GLI1 and GLI2, have been traced to sporadic MB [12]. SHH MB is frequent in infants 0–3 and infrequent in children 3–16 years old [12].

SHH MB occurs across all age groups and exhibits a characteristic bimodal distribution, being most prevalent in infants (<3 years old) and in adolescents or young adults. It is the dominant molecular subgroup in adult medulloblastoma, comprising over 60% of such cases [15]. SHH tumors in infants often exhibit desmoplastic/nodular or medulloblastoma with extensive nodularity (MBEN) histology, which is associated with a favorable prognosis [16]. In contrast, SHH tumors in children and adolescents may display large-cell or anaplastic morphology and frequently harbor TP53 mutations, a genetic alteration associated with markedly worse outcomes and linked to Li–Fraumeni syndrome in some cases. SHH MB displays broad clinical variability depending on age, histology, and genomic status [17].

Cavalli and colleagues further classified SHH MB into four molecular subtypes based on age, cytogenetic profile, and mutational drivers: SHHα, SHHβ, SHHγ, and SHHδ. SHHα tumors primarily affect children and are enriched for TP53 mutations, MYCN and GLI2 amplifications, and large-cell/anaplastic histology, which collectively confer a very poor prognosis. SHHβ and SHHγ are both infant subtypes, but SHHγ has a markedly better outcome [11]. SHHγ tumors are typically genomically quiet and are associated with MBEN or desmoplastic/nodular histology. SHHβ tumors, by contrast, exhibit greater chromosomal instability and worse outcomes than SHHγ tumors. SHHδ tumors occur in adults and are nearly uniformly associated with TERT promoter mutations, often in combination with other SHH pathway drivers like PTCH1 or SMO [18]. Clinically, SHHδ tumors tend to follow a more indolent course, with moderate to favorable outcomes depending on the degree of residual disease and histologic aggressiveness.

In the context of treatment, TP53-mutant SHH MB poses a significant challenge. These tumors often arise in children aged 4–17 and are commonly refractory to standard therapies, leading to their classification as a “very-high-risk” subgroup in consensus frameworks [19]. These findings have led to de-escalated chemotherapy-based protocols in some infant SHH subtypes [20]. These distinctions emphasize the need for refined, age- and genotype-tailored therapeutic strategies for SHH MB, which remains among the most biologically and clinically diverse subgroup of medulloblastoma.

### 2.3. Group 3

While SHH MB is frequent in infants and less common in older children, Group 3 MB typically presents in younger age groups with aggressive clinical features. Group 3 MBs account for roughly a quarter of cases [21]. Occurring mainly in infants and young children, they are rarer in adults [22], with a strong male predominance (approximately 2:1 male-to-female ratio). Group 3 MBs are aggressive, frequently presenting with metastasis at diagnosis (in up to ~50% of cases), and commonly exhibit large-cell/anaplastic histology [22].

Group 3 medulloblastomas are molecularly characterized by the amplification and overexpression of the MYC oncogene in approximately 15–20% of cases. These MYC-amplified tumors are strongly associated with an undifferentiated, highly proliferative phenotype [23]. These tumors often present with structural rearrangements that move oncogenic transcription factors next to enhancers, which are called “enhancer hijacking” events that lead to aberrant overexpression of genes such as GFI1 or GFI1B in subsets of cases [24]. These enhancer hijackings are significant drivers in the tumorigenesis of these MBs by activating growth-promoting transcription outside of canonical signaling pathways. Unlike WNT or SHH MBs, Group 3 tumors have relatively few recurrent point mutations; however, occasional alterations in epigenetic and chromatin regulators are observed. For example, inactivating mutations of SMARCA4 (which encodes a SWI/SNF chromatin remodeling subunit) occur in a minority (11%) of Group 3 cases [25], and recurrent hotspot insertions in the gene KBTBD4 have been identified [26]. Overall, many of these genetic factors may converge to drive a poorly differentiated, high-risk MB. It is important to note that there are multiple developmental pathways that comprise Group 3, and its precise cell of origin remains uncertain, although evidence suggests an origin from early cerebellar progenitor cells [27].

More recent large-scale transcription and methylation analyses have further classified Group 3 tumors into the Group 3α, 3β, and 3γ subtypes. Group 3α tumors tend to occur in infants (under 3 years old), commonly presenting with the loss of chromosome 8q [28]. Group 3β tumors occur more frequently in early childhood, characterized by a high frequency of GF1/GFI1B activation via enhancer hijacking—these tumors also display OTX2 amplification, a growth-promoting transcription factor. Finally, Group 3γ corresponds to the cases with the greatest risk, due to their MYC amplification with undifferentiated large-cell/anaplastic morphology, and thus has the worst prognosis of the three subtypes. It is important to note that Group 3 and Group 4 tumors do not have unified molecular signatures, making them more difficult to distinguish. However, despite recent advances, Group 3 remains the most aggressive MB subgroup, highlighting the urgent need for novel therapeutic strategies.

Some frameworks classify Group 3 and Group 4 into three subtypes each, while analyses such as SJMB03 define eight combined Group 3/4 subtypes. We have clarified this distinction and included a brief explanation in the text.

### 2.4. Group 4

Group 4 MB is the most common molecular subgroup, comprising approximately 35% of all MBs [29]. It typically presents in childhood (often between about 3 and 16 years of age) and can also occur in adolescents; in contrast to Group 3, it is very uncommon in infants [30]. Group 4 shows a marked male predominance, with a ratio of roughly ~4:1 for males to females [22]. Most Group 4 tumors have a classic histopathology, and a high proportion exhibit the cytogenetic hallmark of isochromosome 17q (balanced gain of 17q with loss of 17p), reflecting common large-scale genomic instability in this subgroup [31]. Clinically, Group 4 disease is considered an intermediate-risk category: metastatic presentation is seen in roughly one-third of cases, which is slightly less frequent than in Group 3 [32].

While certain histological patterns and immunohistochemical markers are correlated with molecular subgroups, they are not sufficient for definitive classification. For example, WNT medulloblastomas are typically associated with a classic histology and nuclear β-catenin staining, and SHH tumors often show desmoplastic or nodular histology in infants. However, overlap exists across subgroups, and large-cell/anaplastic morphology can be seen in both SHH and Group 3 tumors. Similarly, β-catenin staining is not entirely specific to WNT activation. Therefore, full molecular profiling, such as DNA methylation analysis or transcriptomic classification, is required to accurately determine subgroup affiliation in clinical and research settings.

Like Group 3, Group 4 comprises heterogeneous drivers with no singular defining pathway for these tumors. Group 4 is characterized by multiple recurrent gene changes that collectively disrupt the normal regulation of developmental genes. Found in a subset of Group 4 MBs, one important feature is the overexpression of the transcription factor PR/SET Domain 6 (PRDM6) [33]. Similarly to Group 3, the overexpression of PRDM6 is typically caused by an enhancer hijacking mechanism (often involving structural tandem duplication near the PRDM6 locus) [34]. Similar enhancer hijackings can activate GFI1 or GFI1B in a smaller fraction of Group 4 tumors (like those in Group 3) [24]. Group 4, along with Group 3, typically displays large copy number changes, which include events such as the loss of chromosomes 8 and 11, and the gain of chromosome 7 [35].

Also important in Group 4 MB is the recurrent disruption of chromatin modifiers and epigenetic regulators, which shows that epigenomic dysregulation plays an important role in its pathogenesis. Loss-of-function mutations in the Lysine Demethylase 6A (KDM6A), Lysine Methyltransferase 2C (KMT2C), and Zinc Finger MYM-Type Containing 3 (ZMYM3) are enriched in Group 4 tumors, indicating convergent epigenomic dysregulation in tumor development [36]. Group 4 tumor cells may depend on changes in epigenetic landscape to sustain a primitive state, as evidenced by these chromatin regulators [37]. Mutations in X-linked genes like KDM6A and ZMYM3 may somewhat explain the male predominance of Group 4, as a single hit has the capability to disable these genes in males [38]. As a whole, Group 4 MBs seem to arise from the dysregulation of developmental transcriptional programs and chromatin, driven by a combination of structural variants and mutations. WNT medulloblastomas are thought to originate from progenitor cells of the lower rhombic lip or dorsal brainstem, aligning with their midline anatomic presentation and low metastatic potential. SHH medulloblastomas are believed to arise from granule neuron precursors in the external granule layer of the cerebellum, consistent with their frequent desmoplastic histology. Group 3 and Group 4 tumors are less well understood but are hypothesized to arise from early cerebellar stem-like progenitor cells or uncommitted neural precursors [39]. Single-cell transcriptomic analyses suggest that Group 3 tumors may originate from Nestin+ neural stem cells with *Myc*-driven transcriptional programs, while Group 4 may stem from more differentiated glutamatergic lineage precursors [28]. Despite progress in mapping these developmental origins, the precise lineage relationships remain an active area of research [27].

The heterogenous Group 4 has also been further subdivided into the following subsections: Group 4α, 4β, and 4γ. Group 4α is enriched with MYCN amplifications. On the other hand, 4β tumors are marked by the presence of SYNCAIP tandem duplications and, oftentimes, the activation of PR/SET Domain 6 or GFI1/B. Finally, 4γ has CDK6 amplification with a loss of chromosome 8p and the gain of chromosome 7q. Group 4 subtypes were treated similarly in the clinic based on risk stratification, despite genetic differences, however. Though Group 4 presents an intermediate prognosis, the molecular heterogeneity within this subgroup underscores the necessity of further personalized therapeutic approaches. Table 3 summarizes the comparisons between the classification of MB subtypes from 2012 by Taylor et al. [12] and from 2017 by Cavalli et al [11].

## 3. Standard Treatment: Surgery, Radiation Therapy, and Targeted Therapies

Standard treatment for medulloblastoma has long consisted of maximal safe resection followed by risk-adapted craniospinal irradiation (CSI) and multi-agent chemotherapy. However, the specifics of radiation dose and chemotherapy regimens are stratified by the patient’s risk category and age [40]. Current protocols distinguish average-risk vs. high-risk disease to tailor therapy intensity:

Average-Risk (Standard-Risk) Patients: These are typically defined as children ≥3–4 years old with no metastases (M0) and near-total resection of the tumor (≤1.5 cm^2^ residual). Standard management includes CSI at a reduced dose of 23.4 Gy, followed by a boost of up to ~54 Gy to the tumor bed or posterior fossa. Current trials exploring further de-escalation (e.g., in WNT MB) are evaluating reductions to 15–18 Gy [40]. This lower CSI dose is used to limit neurotoxicity while still providing prophylactic treatment to the entire CNS. After radiation, patients receive adjuvant chemotherapy, often a regimen such as weekly vincristine during RT and then multi-cycle “Packer regimen” (cisplatin, lomustine, vincristine), or analogous combinations. Using this approach, 5-year survival in average-risk medulloblastoma now exceeds 80% [41]. Notably, even within the “average-risk” category, emerging biological research can further stratify therapy: for example, WNT subgroup patients (who almost all fall into average-risk clinically) display such favorable outcomes that recent trials (e.g., SIOP PNET5 MB) have tested CSI dose reductions and/or omitted certain chemotherapeutic agents for WNT patients. The rationale is to maintain their ~95% survival while minimizing long-term side effects.

High-Risk Patients: Patients with metastatic disease (M+) and/or residual tumor >1.5 cm^2^, sometimes defined to include very young patients or unfavorable biology, are treated more aggressively. However, very young infants typically do not receive CSI due to neurotoxicity risks. Instead, they are treated with high-dose chemotherapy with autologous stem cell rescue (HDCT/AuSCR). CSI is only considered in cases of relapse or treatment failure. For older high-risk patients, CSI is given at a higher dose followed by boosts to gross disease [40]. The chemotherapy is also intensified; high-risk protocols often add agents like methotrexate or high-dose chemotherapy with stem cell rescue. For example, the COG ACNS0332 trial for high-risk MB tested intensification with carboplatin during radiation and added intravenous methotrexate cycles—an approach targeting MYC-amplified or metastatic cases especially. Five-year survival for high-risk patients is in the order of ~60–70% with these intensified regimens [41]. Given the poorer prognosis, high-risk patients may also be candidates for experimental therapies (e.g., targeted inhibitors in protocol settings) to improve outcomes. Importantly, molecular features are now influencing risk stratification in clinical practice: for instance, SHH subgroup tumors with TP53 mutations are sometimes treated using separate “very-high-risk” protocols (with upfront novel agents or transplant) because of the historical <50% survival rate despite maximal standard therapy use [40]. Similarly, a patient with Group 3 medulloblastoma harboring MYC amplification would be considered high risk by most groups, even if non-metastatic, and therapy might be augmented (some protocols recommend autologous stem cell transplant or targeted trials for this subset).

Young Children (<3 years): Infants and toddlers represent a special therapeutic category. Standard CSI is contraindicated due to devastating neurodevelopmental toxicity in the developing brain [42]. Thus, radiation-sparing approaches are used: surgery followed by intensive chemotherapy-only regimens, often including high-dose chemotherapy with stem cell rescue (e.g., the “Head Start” protocols). These regimens achieve remission in a proportion of young patients, especially those with desmoplastic histology (often SHH-MB infant subtype) which respond well to chemotherapy [42]. If relapse occurs or if disease is high risk (e.g., metastatic infant MB), some children receive delayed CSI at a reduced dose once they are a bit older. Overall survival in children < 3 years old has historically been lower, but outcomes are improving with modern protocols. Notably, molecular stratification is being applied here as well: infants in the SHH subgroup or showing desmoplastic medulloblastoma have excellent chemo-only survival (~75% in some series) and may avoid any radiation, whereas infants in Group 3 (especially if MYC-driven) have poor outcomes with chemotherapy alone, prompting the consideration of novel therapies. The COG ACNS0334 study demonstrated important subgroup-specific outcomes, particularly for SHH MBs [43]. In contrast, the SJYC07 study from St. Jude showed poor survival among infants with Group 3 tumors [44]. These studies provide necessary nuance to therapeutic expectations in young children.

Rationale for CSI dosing: The dose of craniospinal irradiation is a cornerstone of risk-adapted therapy. Lowering CSI from ~36 Gy to 23.4 Gy in the 1990s was a breakthrough for average-risk patients, significantly reducing neurocognitive sequelae while maintaining survival at ~80% [45]. On the other hand, high-risk patients require the full-dose CSI to achieve adequate disease control given their higher burden and aggressiveness of disease [40]. Ongoing studies aim to make radiation-based approaches more personalized: for instance, if molecular profiling identifies a truly radiosensitive tumor (such as WNT), future treatment protocols may allow for de-escalated CSI in the context of clinical trials. Conversely, if a tumor is biologically high-risk, some advocate augmenting local doses or adding focal boosts to metastatic nodules. The guiding principle is to maximize curative properties while minimizing harm, using risk factors to avoid overtreatment in low-risk patients and under treatment in high-risk cases.

As such, standard therapy is no longer one-size-fits-all—instead, it is stratified by clinical risk group and increasingly by molecular subtype. Average-risk/WNT patients are candidates for therapy reduction to improve quality of life, whereas high-risk or molecularly high-risk patients receive intensified regimens in order to attempt to improve survival. This risk-adapted paradigm underpins current treatment algorithms and clinical trial designs worldwide, where multifaceted treatment strategies including surgery, radiation therapy, and chemotherapy must be carefully tailored to individual patient profiles. Treatment decisions for pediatric embryonal tumor cases are based on the age of the child, progression, genetic predisposition, and pathological MB type. Treatments include surgical intervention, radiation therapy (if over 3 years), and targeted therapies. Generally, the five-year survival rate for pediatric patients with standard-risk MB is 75–90 percent, while the rate for pediatric patients with high-risk MB can range from 50 to 75 percent, depending on clinical and molecular risk factors [17].

The WNT subgroup prognosis is highly favorable (discussed further under the title ‘Outcomes Based on Molecular Subtypes’). While approximately 10% of WNT MB patients present with metastatic disease, these cases are often still curable with current therapies, including craniospinal irradiation. The prognosis of pediatric patients with SHH subtype MB is more variable and is particularly reduced in the presence of TP53 mutations, which are associated with high-risk disease. In SHH MBs, 10-year survival rates are approximately 77% in infants and 51% in older children, with a modest incidence of metastasis [17]. Metastasis is more common in non-WNT/non-SHH tumors (Group 3 and Group 4), where ~50% of Group 3 and ~30% of Group 4 patients present with disseminated disease. Group 4 tumors are considered intermediate-risk subtypes, with 5-year survival rates generally estimated between 75 and 80%, though some low-risk subtypes may fare better. In contrast, Group 3 has the poorest prognosis, with 10-year survival rates of approximately 39% in infants and 50% in pediatric patients, due in part to frequent MYC amplification, large-cell/anaplastic histology, and aggressive metastatic presentation [17,46].

### 3.1. Surgical Interventions

In the case of MB, surgical intervention is individualized to the tumor and the history of treatment/symptoms. At the present time, surgery is not individualized to MB molecular groups. Generally, pediatric surgical considerations mimic those of adult surgical management. Such surgical intervention aims to maximize tumor removal and minimize neurological damage. Among MB resection cases, approximately 25% of patients develop a degree of cerebellar mutism (Posterior Fossa Syndrome; PFS), resulting in irritability, reduced ability to produce words, and the development of hypotonia and ataxia manifesting 24 h after the surgery, often lasting months with a resulting lifelong difficulty with language [47]. PFS has recently been categorized into type 1 and type 2, noting complete (PSF1) or partial mutism (PFS2) [48]. A preference in surgical approach, whether telovelar or transvermian, has not been proven to impact PFS outcomes.

As MB often grows in the midline of the cerebellum, most MB patients also present with intracranial pressure as a result of obstructive hydrocephalus, manifesting in long-term headaches, nausea, irritability, poor feeding, and extremely altered mental status in children. Possible surgical interventions for obstructive hydrocephalus include cerebrospinal fluid diversion or insertion of an external ventricular drain. When permanent CSF diversion is needed, an endoscopic ventriculostomy (ETV) or a ventriculoperitoneal shunt is performed, allowing fluid to bypass the obstructed posterior fossa [49]. A common complication of surgical management such as this is the risk of infection. An awareness of potential complications such as cerebellar mutism and infection risk informs careful surgical planning and postoperative management strategies.

### 3.2. Chemotherapy and Radiation Treatments

There have been several clinical trials stratified in relation disease subgroups with various outcomes. Although an exhaustive review of current clinical trials is beyond the scope of this review, we will highlight several trials that have shown promising results.

a. ACNS0331

This trial randomized children with average-risk disease to standard dose (23.4 Gy) or reduced dose (18 Gy) CSI with adjuvant chemotherapy, including cisplatin, lomustine, and vincristine, alternating with cyclophosphamide and vincristine. Benefits were seen only in WNT-activated subgroups [50].

b. ACNS0332

This trial assessed the addition of carboplatin concomitantly with radiation and the role of isotretinoin as a pro-apoptotic agent in newly diagnosed high-risk medulloblastoma. Subgroup analysis showed a survival advantage with the addition of carboplatin to radiation in Group 3 MB patients [51].

c. ACNS0334

This trial assessed high-dose chemotherapy in high-risk group young children using three cycles of intensive chemotherapy randomized to high-dose methotrexate vs. no methotrexate, followed by three cycles of high-dose carboplatin and thiotepa with stem cell rescue. Patients treated with methotrexate demonstrated a better 5-year progression-free survival [43].

d. Head Start III

This trial used highly intensive treatment regimens including methotrexate (ACNS0334), followed by one cycle of high-dose thiotepa, carboplatin, and etoposide. Radiation was reserved for children 6 years and older or those without complete response. Patients with nodular and/or metastatic desmoplastic disease had the best outcomes, suggesting that traditional risk staging can be overcome by high-dose chemotherapy regimens [52].

e. Head Start IV

This is a prospective randomized study based on molecular subtypes and response to induction chemotherapy (vincristine, cisplatin, cyclophosphamide, etoposide, and high-dose methotrexate), allowing to compare the efficacy of one vs. three tandem cycles of myeloablative therapy. Children with localized SHH MBs had a 96% 3-year event-free survival and 100% overall survival. Estimated 3-year event-free survival rates for SHH subtype 1 and 2 patients were 100% and 95%, respectively [53].

f. SJMB03

This trial was a risk-adaptive trial for average and high-risk disease. CSI dosing was reduced to 23.4 Gy in average-risk patients and 36–39 Gy in high-risk patients. Four cycles of adjuvant high-dose chemotherapy were given with stem cell support. WNT subtype patients had excellent 100% 5-year event-free and overall survival. Poor outcomes were seen in patients in the SHH subgroup, patients with metastatic disease, *MYCN* or *GLI12* amplification, or chromosome 17p loss [40].

Taken together, these clinical trials demonstrate promise if MB patients are properly stratified across subgroups with intensive upfront chemotherapy regimens. For ongoing trials and other treatment strategies including strategies at relapse, we refer our readers to an excellent review by Dr. Michael Prados [54].

### 3.3. Molecularly Targeted Therapies: SHH Subgroup, WNT Subgroup, Non-SHH/WNT Subgroup

#### 3.3.1. SHH

**Figure 2:**
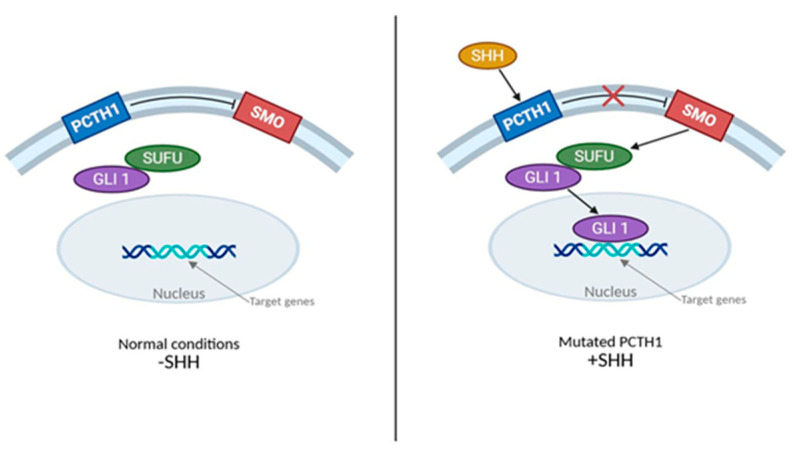
PTCH1 Targeted therapy for treatment of SHH subgroup Medulloblastoma through SMO inhibition. A figure depicting normal Hedgehog signalling and SMO inhibitor action in Medulloblastoma. −SHH. Hedgehog proteins effectively bind to PTCH1 transmembrane protein and stops inhibition of SMO pathway. The now active SMO migrates to cilium and activates release of suppressor of fused (SUFU) inhibition of glioma-associated oncogene (GLI) proteins that then translocate to the nucleus to affect transcription of SHH target genes (e.g., PTCH1). +SHH. Pathway is inhibited by SMO inhibitors, preventing pathway signalling and activation of SMO.

As knowledge of molecular classifications and biological heterogeneity of MB has increased, molecularly targeted therapies (i.e., precision therapy) have been developed to target the genetic changes associated with the different subtypes of MB in children as classified by WHO CNS5. The most widely studied targeted therapies for combating the SHH subgroup are SMO inhibitors such as HhAntag, vismodegib, saridegib, and sonidegib, use to target the binding of Sonic Hedgehog to its receptor Patched 1 (PTCH1), which activates downstream signaling via the mediator smoothened (SMO) [4]. Vismodegib and other SMO inhibitors can cause the premature fusion of the growth plates; hence, their use is restricted to adolescents and young adults who are skeletally mature (Figure 2).

#### 3.3.2. WNT

**Figure 3:**
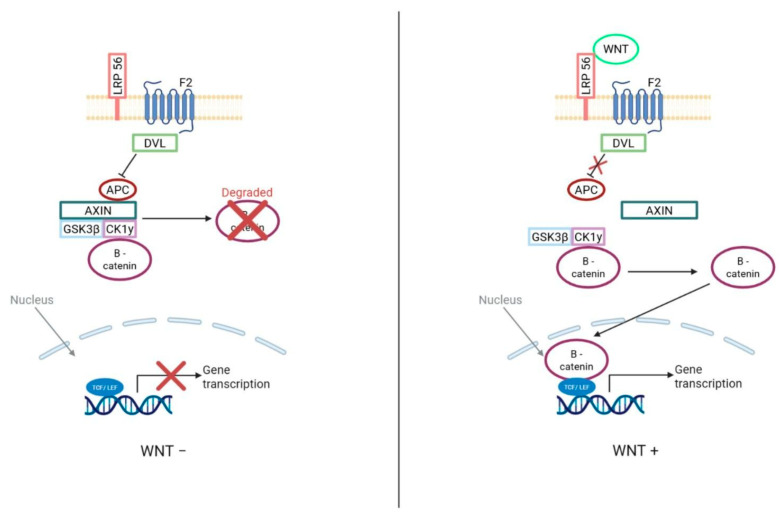
WNT Signalling Pathway. Targeted therapy for treatment of WNT subgroup Medulloblastoma through b-catenin inhibition. A figure depicting protein phosphatase inhibition in active and inactive WNT signalling pathways. −WNT. Without the presence of WNT, b-catenin is phosphorylated by the protein complex leading to proteasomal degradation. A lack of b-catenin in the nucleus leads to the binding of a TCF/LEF repressor complex to repress gene activity. +WNT. As WNT binds to the Frizzled and LRP co- receptors, LRP receptors receive phosphorylation and enlist DVL proteins to provoke the disassembly of the protein complex responsible for the degradation of b-catenin; Resulting in a surplus of b-catenin in the cytoplasm and allowing it to translocate to the nucleus, forming a complex with TCF/LEF to transcribe target genes.

Given the genetic drivers discussed previously (CTNNB1 mutations, APC alterations, and Turcot syndrome), new targeted therapies aim to inhibit downstream signaling mediated by β-catenin, specifically targeting Cyclin D1 and MYC. The purpose of the new drugs is to combat tumors of the WNT subgroup, targeting the downstream signaling by beta-catenin of Cyclin D1 and MYC [4]. These drugs include naturally occurring protein phosphatase inhibitors, cantharidin, norcantharidin, and ginkgetin. Due to the better prognosis of patients in the WNT subgroup, treatments are generally aimed at limiting cytotoxic therapies (Figure 3) [4].

#### 3.3.3. Non-WNT/SHH

As Group 3 non-WNT/SHH tumors are often characterized by their MYC amplification, targeted therapies are being developed for downstream targets of MYC [55]. Due to a lack of knowledge regarding the signaling pathways implicated in non-SHH/WNT targeted therapeutics have not yet been developed for Group 4 of this subtype.

## 4. Outcomes Based on Molecular Subtypes

This section focuses on clinical outcomes and recurrence patterns based on molecular subgrouping, drawing on large cohort studies and clinical trial data. Clinical outcomes are intrinsically linked to the molecular subtypes and the tailored treatments discussed previously, emphasizing the importance of precision medicine. The molecular subclassifications of MB indicate that outcomes significantly vary between subgroups. Below, we summarize survival rates, recurrence patterns, and key prognostic indicators for each molecular subtype, incorporating the data from clinical trials and genomic studies.

### 4.1. WNT Subgroup

The WNT-activated MBs account for about 10% of MB cases, having the most favorable prognosis among all MB subtypes with a 5-year survival rate of over 95% [56,57]. The WNT subgroup responds well to chemotherapy and radiation, and rarely relapses after application of standard risk-adapted therapy [32]. Now, late toxicity and secondary malignancies account for significant morbidity and mortality, rather than tumor recurrence [58]. Some key markers of this subtype include activating mutations in CTNNB1 (β-catenin) and monosomy 6, with nuclear β-catenin positivity being both diagnostically and prognostically favorable [59]. Specifically, WNT tumors have a low rate of metastasis (less than 5% of patients) and a favorable prognosis, even in metastasis events [60]. Currently, clinical trials are exploring reduced-intensity therapy to minimize long-term treatment-related toxicity for this subgroup [61].

### 4.2. SHH Subgroup

SHH-activated MBs account for 25 to 30% of MB cases and have intermediate outcomes, notably influenced by TP53 status [21]. SHH typically has a 5-year survival rate of 75 to 85%, which is similar to the rate of overall average-risk MB [30,31]. However, the presence of a TP53 mutation significantly worsens prognosis, with 5-year survival rates often dropping below 50% [62]. These tumors frequently present with large-cell/anaplastic histology and have an early relapse, which prompts high-risk experimental protocols. On the other hand, TP53-wildtype SHH tumors typically lead to better outcomes than TP53-mutant tumors; however, genetic features like MYCN or GLI2 amplification can significantly worsen the trajectory. The five-year survival rate for TP53-wildtype SHH cases is approximately 75% and even lower with MYCN or GLI2 amplification—comparatively, TP53-mutant SHH tumors have survival rates of less than 50%. Infants with desmoplastic/nodular or MB with extensive nodularity (MBEN) histology generally have favorable outcomes using chemotherapy protocols, whereas those with non-desmoplastic or TP53-mutant SHH tumors tend to have worse outcomes [42]. In terms of treatments, targeted therapies such as SMO inhibitors (e.g., sonidegib or vismodegib) may be promising in treating recurrent adult SHH tumors, although concerns about toxicity are preventing pediatric application [63].

### 4.3. Group 3 MB

Group 3 tumors represent 20 to 25% of MB cases and historically have the poorest prognosis [64]. Group 3 frequently presents with metastases (at a rate of about 50%) and has large-cell/anaplastic histology [65]. Overall, 10 to 17% of cases also have an amplified MYC oncogene, significantly worsening prognosis [66]. On the other hand, non-MYC-amplified, non-metastatic Group 3 tumors have higher survival rates (around 60%) [67]. However, relapse (typically early and metastatic) is common and predominantly occurs via leptomeningeal dissemination [68]. The SJMB03 trial recently subclassified group 3/4 into eight subtypes, identifying notably poor outcomes (~33% survival rate) for Subtype III (MYC-amplified, infants); this highlights the need for novel treatments [40]. Therapeutically intensified regimens (such as carboplatin radiosensitization and high-dose chemotherapy) remain standard for high-risk Group 3, while newer approaches like immunotherapy and targeted molecular agents are actively investigated [69]. The identification of high-risk features such as MYC amplification underscores the need for intensified and targeted therapeutic approaches.

### 4.4. Group 4 MB

Group 4 is the most common subgroup (representing roughly 35%) of MB cases, having an intermediate prognosis with a 5-year survival rate of approximately 75%, although outcomes vary significantly by molecular characteristics [29]. Certain subsets identified by recent subclassification (e.g., Subtype VII in the SJMB03 trial, ~92% 5-year progression-free survival) typically feature chromosome 11 loss or specific genetic alterations (such as KBTBD4 mutations), which leads to better outcomes [29]. On the other hand, MYCN-amplified tumors or those with metastatic disease have worse outcomes. Group 4 tumors tend to relapse later in life and with lower frequency than Group 3, and recurrence is often with mixed local and spinal metastases [17]. Current therapies have focused on managing risk stratification to appropriately intensify or de-escalate treatment; however, specific targeted therapies remain unavailable, leading to a reliance on conventional treatments [54].

**Figure 4:**
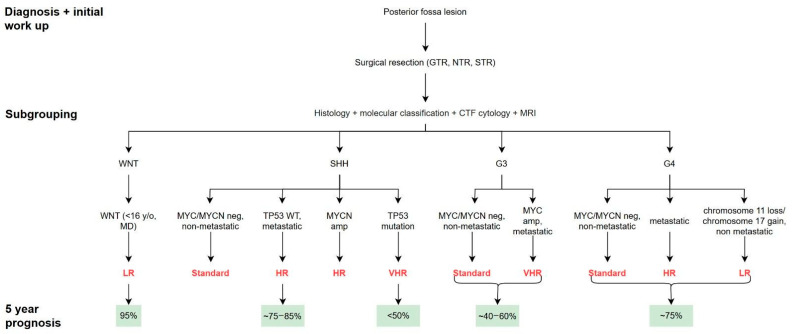
Medulloblastoma Outcomes Flowchart. A figure depicting the typical outcome per subtype of medulloblastoma and prognosis.

While overall survival varies by molecular subtype, long-term quality of life—particularly neurocognitive function—remains a major concern for survivors of pediatric medulloblastoma. Proton radiotherapy (PRT) has emerged as a treatment modality that significantly reduces radiation exposure to normal brain tissue due to its superior dose distribution. In a longitudinal cohort study by Kahalley et al., children treated with PRT demonstrated stable trajectories in overall IQ, working memory, and perceptual reasoning, in contrast to those receiving conventional photon radiotherapy (XRT), who experienced significant declines across these domains [70]. Notably, although both groups showed declines in processing speed over time, the rate of decline was significantly attenuated in the PRT group. These findings highlight the cognitive benefits of PRT and have contributed to its increasing adoption in treatment planning—particularly for younger patients at elevated risk of long-term neurocognitive sequelae due to CSI (Figure 4).

## 5. The Future of Medulloblastoma Treatment

Building upon current treatment paradigms, recent insights into MB biology have ushered in the use of novel therapeutic strategies aimed at increasing efficacy and reducing toxicity. Treatment for medulloblastoma is a rapidly evolving landscape as new insights into the tumor biology of these malignancies are driving the development of more effective targeted therapies and veer away from non-specific systemic chemotherapies. One of the more promising areas in medulloblastoma treatment is the development of targeted therapies such as CT179, a novel inhibitor of OLIG2, a helix–loop–helix (HLH) transcription factor that is known to be essential in maintaining tumor cells in certain medulloblastoma subtypes. In preclinical studies, it has been shown that when pairing CT179-mediated OLIG2 inhibition with CDK4/6 inhibition, there is a synergistic effect and the significant suppression of tumor growth [71]. CT179 is one of many small-molecule inhibitors that have shown promise in treating this malignancy and highlights a potential role for these agents in treatment, as the understanding of medulloblastoma biology continues to become more understood. Similarly, the alterations to chromatin and transcriptional landscape in *MYC*-amplified MBs lend well to the antitumor effects of histone deacetylase (HDAC) inhibitors such as panobinostat and vorinostat, and to inhibitors of intracellular signaling PI3K/AKT pathways, such as GDC-0941 and BKM120, that are associated with enhanced tumor growth, metastasis, and chemoresistance [72]. Finally, an emerging class of inhibitors targeting neurotrophin TrkB receptors has demonstrated antitumor effects and shown that elevated levels of neurotrophin 1 or 2 receptors are associated with reduced overall survival in SHH MB patients, hinting at the therapeutic target of this group of growth receptors [73].

In recent years, immunotherapy has been shown to have a powerful role in combating pediatric low-grade and high-grade gliomas; however, the role for this approach in medulloblastoma treatment is now actively being explored [74,75]. Recent work has shown that SHH MBs have significantly higher protumorigenic tumor-associated macrophages (TAMs), higher percentages of dendritic cells, infiltrating lymphocytes, and myeloid-derived suppressor cells than Groups 3 or 4 MBs, with Group 3 MBs having a higher percentage of CD8+ T cells [76,77]. Bockmayr and colleagues showed that SHH MBs have strong fibroblast, T cell and macrophage signatures, with cytotoxic lymphocytes being enriched in Group 4 MB [78]. Interestingly, Maximov and colleagues reported an antitumor TAM population in SHH MBs [79], and Yao et al. reported that tumor-derived astrocytes secrete IGF1 to polarize the TAMs to promote tumor progression. Furthermore, the co-expression of *Myc* and dominant-negative *Trp53* led to tumor suppression in immunocompetent mice due to rejection by T cells, while tumor necrosis factor could overcome immune evasion in *Myc*-overexpressing and dominant-negative *Trp53* MB cells, demonstrating the potential for manipulating the immunosuppressive tumor microenvironment as a therapeutic target for the treatment of MBs. Single-cell analysis of MB tumors identified clusters of lymphocytes and myeloid cells, with M2-activated myeloid cell enrichment seen in SHH MBs compared to Group 3 and 4 MBs [80]. Finally, Dang and colleagues identified distinct subpopulations of TAMs that were derived from monocytes and microglia that could be regulated by chemo- and radiation therapy, suggesting that the ability to manipulate the tumor immune microenvironment towards a pro-inflammatory, pro-immune state could increase the efficacy of existing and novel treatments [81].

Given the recent success in treating diffuse midline glioma (DMG) with GD2 CAR-T cell therapy, as well as promising preclinical findings for using this approach in medulloblastoma, researchers are now exploring whether GD2 CAR-T cell therapy is effective in combating GD2+ medulloblastoma [82,83,84]. Other CAR-T targets include HER2, B7-H3, EPHA2, GD2, PRAME207, and IL-13 receptor α2, which are also expressed on the surface of MB tumor cells [85,86,87]. Furthermore, more broad immunotherapy approaches for medulloblastoma are also being explored, such as natural killer (NK) cell therapy, oncolytic viruses, as well as cancer vaccines and immune checkpoint inhibition [83,88,89]. While the CNS does present unique challenges for immunotherapy such as the BBB and localized immunosuppression, advances in cell engineering as well as immunotherapy delivery and combinatorial treatment are aimed at circumnavigating these barriers.

Finally, the treatment of medulloblastoma using metronomic dosing of chemotherapy, as well as alternative chemotherapies, for recurrent or high-risk diseases has also been gaining traction in recent years. Specifically, it was shown that when alternating standard-of-care chemotherapy with lose-dose (metronomic) oral etoposide and cyclophosphamide supplemented with intravenous bevacizumab, there was an overall five-year progression-free survival of 49.7% [90]. Furthermore, the addition of carboplatin (a radiosensitizer) to medulloblastoma treatment protocols was shown to improve event-free survival by 19% at five years in children with high-risk Group 3 disease. Treatment with isotretinoin, which was evaluated concurrently in a separate arm of the study, was determined to be clinically futile and was discontinued mid-study by the Data Safety Monitoring Committee (DSMC) [51].

Several challenges exist in defining biomarker validation and obtaining research biopsy procurement in pediatric neuro-oncology trials. Characterizing the early-phase pharmacokinetic and the pharmacodynamic efficacy of novel targeted therapies improves the chances of FDA approval [91]; however, there remains ethical considerations in procuring research biopsies in the vulnerable and pediatric population with the need for open communication and expectations between the parents and the providers as to the risks and benefits of obtaining serial research biopsies throughout the course of a MB or other pediatric neuro-oncology clinical trial [92,93]. More recently, the technological advancements of being able to sequence and characterize circulating tumors cells and cell-free tumor DNA in the CSF represent viable alternatives in obtaining samples that recapitulate the genomic alterations present in the tumor (including mutations, CNVs, chromosomal gains and/or losses) [94,95]. This less invasive alternative may offer a solution to the ethical controversies of the need for obtaining serial research tissue biopsies in this patient population.

Taken together, these developments underscore a paradigm shift in the treatment of medulloblastoma, away from uniform high-toxicity regimens and toward individualized, biologically informed treatment strategies. The integration of small-molecule inhibitors such as CT179, as well as the development of innovative immunotherapeutic approaches, such as CAR-T cell therapy and vaccines, and the promise of metronomic chemotherapy dosing, represent a promising new frontier for medulloblastoma treatment. As clinical trials advance these innovative therapies, the hope is not only to improve patient survival but also to significantly enhance quality of life through precision medicine. Table 4 summarizes new avenues of therapies for the treatment of MB.

### Global Disparities and Translational Challenges

While advancements are being made in terms of molecularly focused therapies, it has also called into question how these advancements would be implemented globally, and in an equitable manner. There continue to be vast disparities in outcomes and technological access between high-income and low/middle-income countries (LMICs): while children with average-risk MB in well-resourced centers now have an >80% 5-year survival, less-funded areas have survival rates that lag far behind—oftentimes only 50–60% or less [41,96]. Namely, there are a few key factors that contribute to this gap.

First, access to molecular diagnostics is limited in much of the world: valuable tools like DNA methylation profiling or panel sequencing require expertise and infrastructure often lacking in hospitals in LMICs. This prevents problems from being identified in the first place [96]. In light of this problem, there are efforts to create cost-effective molecular tests, such as simplified IHC or PCR-based panels, to help distinguish WNT or SHH tumors when resources are limited [97]. 

Furthermore, international collaborations such as St. Jude’s Global program in 2020 have also been helping establish central referral labs for analysis to serve under-resourced hospitals (https://global.stjude.org/en-us/programs/transversal-programs/neuro-oncology.html (accessed on 6 July 2025). Overcoming this division in diagnostic accessibilities is imperative in ensuring that integrated risk stratification and modern trial strategies can be applied to all patients, rather than only a small subset in wealthy countries.

Aside from diagnostics, another critical challenge is access to therapies, especially those that are newer or more expensive. Many essential chemotherapy drugs are intermittently available or cost-prohibitive in low-income regions [97]. Furthermore, introducing novel agents like SHH pathway inhibitors can be very costly: for instance, an SHH inhibitor (like vismodegib) may show promise for relapsed cases of SHH-MB, but this drug might be inaccessible outside of a trial due to costs. Even conventional therapies, like pediatric radiotherapy, are low in supply as many countries lack proton therapy centers and even linear accelerators may be scarce in certain areas [96]. As such, the resource gap realistically must be addressed so that MB patients worldwide can achieve the maximal benefit from these theoretical outcomes.

While the future in treating MB is hopeful, with precision stratification and new therapies in the near future, the goal of curing every child afflicted with MB will not only require scientific innovation but a shared effort to overcome practical challenges, including cost, access, and infrastructure. Risk-based therapy, when implemented equitably, is necessary to ensure that robust advancements in trials can reliably translate into survival for children around the world.

## Figures and Tables

**Table 1:** Number of Cases of Medulloblastoma and subtypes from 2017/2018–2021 in the United States of America.

	Total # Cases 2017–2021	Age Range
Medulloblastoma	1395	0–14
	**Total # Cases 2018–2021**	**Age Range**
SHH Subtype	397	5–31
WNT Subtype	74	8–14
Non-SHH/WNT	295	8–12

**Table 2:** Comparison summary of WHO CNS5 Medulloblastoma classification updates vs WHO CNS4 updates. Compares the 2016 WHO CNS4 classification and the later 2025 WHO CNS5 classification of medulloblastoma.

WHO CNS5 2021 Medulloblastoma Classification Overview		
	WHO CNS4 (2016)	WHO CNS5 (2021)
Knowledge of Genes/Molecular Profiles Characteristically Altered	- WNT-activated - SHH-activated - Non-WNT/non-SHH (Group 3/Group 4)	WNT-activated: CTNNB1, APC SHH-activated: TP53, PTCH1, SUFU, SMO, MYCN, GLI2 (methylome) - Four Subtypes Non-WNT/non-SHH: MYC, MYCN, PRDM6, KDM6A (methylome) - 8 Subtypes
Morphology	- Classic - Desmoplastic/nodular - Medulloblastoma with extensive nodularity (MBEN) - Large cell/anaplastic	Combined into 1 section (Medulloblastoma, histologically defined) that describes them as morphologic patterns of an inclusive tumor type. Most Common Associations: - The majority of WNT-activated MBs are linked = classic morphology - Majority of large cell/anaplastic tumors = SHH-3 subgroups or G3/4 subgroup 2
Remaining the same	- Histopathological classification - CNS WHO grade 4 designation of Medulloblastoma	
New	- Guidance on distinguishing tumor subtypes through molecular markers instead of histology - Stratifying pediatric brain tumors by genomic and epigenomic features	

**Table 3:** Comparison of the 2012 Taylor et al. [12] and 2017 Cavalli et al. [11] medulloblastoma classification systems. Compares the four major molecular subgroups in terms of subgrouping, molecular features, and clinical relevance.

Subgroup	2012 Taylor et al. Classification of MB [12]	2017 Cavalli et al. Classification of MB [11]
WNT	Single subgroup characterized by CTNNB1 mutations and monosomy 6. Excellent prognosis.	Split into WNTα (pediatric, monosomy 6) and WNTβ (older patients, no chr6 loss). Both have excellent outcomes.
SHH	Single SHH subgroup, known age bimodality (infants & adults). TP53 mutation linked to poor outcome.	Refined into 4 subtypes: • SHHα (children, TP53 mutations, poor prognosis) • SHHβ (infants, poor outcome + frequent copy number alterations) • SHHγ (infants, desmoplastic/nodular, good prognosis, MBEN-enriched, genomic quietness) • SHHδ (adults, TERT mutations)
Group 3	Defined as aggressive, MYC-amplified, high metastasis. Poor prognosis.	Refined into 3 subtypes: • Group 3α (infants, 8q-loss + lack major driver amplifications) • Group 3β (GFI1/GFI1B-driven) • Group 3γ (MYC-driven, worst prognosis)
Group 4	Most common subgroup; less well understood. Isochromosome 17q frequent. Intermediate prognosis.	Refined into 3 subtypes: • Group 4α (MYCN, CDK6 amplification) • Group 4β (SNCAIP tandem duplication) • Group 4γ (8p loss)

**Table 4:** Summary of the new therapeutic directions mentioned. Table summarizing the therapeutic directions of medulloblastoma. Categorizes targeted therapies, immunotherapy, and metronomic dosing of chemotherapy.

Summary of New Therapeutic Directions	
Targeted Therapies	CT179: A novel small molecule inhibitor of OLIG2 (a helix-loop-helix (HLH) transcription factor), a key factor in maintaining tumor cells in certain medulloblastoma subtypes. Pairing of CT179-mediated OLIG2 inhibition with CDK4/6 inhibition has had a synergistic effect and significant suppression of tumor growth (63).
Immunotherapy	Powerful role in combating pediatric low-grade and high-grade gliomas; use is now being explored in Medulloblastoma treatment.
	GD2 CAR-T cell therapy: Recent success in treating diffuse midline glioma (DMG) with GD2 CAR-T cell therapy. Preclinical findings show promise for using this approach in combating GD2+ medulloblastoma (66–68).
	Natural Killer (NK) cell: A broader immunotherapy approach is being explored.
	Challenges for immunotherapy: The blood-brain barrier (BBB) and localized immunosuppression.
Metronomic Dosing of Chemotherapy	It has been shown that low-dose (metronomic) oral etoposide and cyclophosphamide supplemented with intravenous bevacizumab has led to an overall five-year progression-free survival of 49.7% (71).
	Addition of carboplatin (a radiosensitizer): Shown to improve event-free survival by 19% at five years in children with high-risk Group 3 disease.

## Data Availability

Not applicable.
